# Supplementary material for: 6′-O-Caffeoylarbutin from Quezui Tea: A Highly Effective and Safe Tyrosinase Inhibitor
Source: Int J Mol Sci. 2024 Jan 12;25(2):972. doi: 10.3390/ijms25020972 (PMC10816276; doi:10.3390/ijms25020972)
Supplement: Supplementary file 1 [file ijms-25-00972-s001.zip › ijms-2746276-supplementary.pdf]

6'-*O*-Caffeoylarbutin from Quezui Tea: A Highly Effective and Safe Tyrosinase Inhibitor

Supplementary Materials

Dong Xie [1,†], Wengan Fu [1,†], Tiantian Yuan [1], Kangjia Han [1], Yuxiu Lv [1], Qi Wang [1], Qian Jiang [1], Yingjun Zhang [2], Guolei Zhu [1], Junming Xu [3], Ping Zhao [1,*] and Xiaoqin Yang [1,*]

[1] Laboratory of National Forestry and Grassland Administration on Highly-Efficient Utilization of Forestry Biomass Resources in Southwest China, Southwest Forestry University, Kunming 650224, China; dong-xie@outlook.com (D.X.); 18751856651@163.com (W.F.); 19387322404@163.com (T.Y.); xqyhankangjia6174@163.com (K.H.); lvyuxiu0130@outlook.com (Y.L.); qiwang202312@163.com (Q.W.); m17368708632@163.com (Q.J.); guoleizhu@163.com (G.Z.)
[2] Laboratory of Phytochemistry and Plant Resources in West China, Kunming Institute of Botany, Chinese Academy of Sciences, Kunming 650204, China; zhangyj@mail.kib.ac.cn
[3] Institute of Chemical Industry of Forest Products, Chinese Academy of Forestry, Nanjing 210042, China; xujunming@icifp.cn
* Correspondence: hypzhao2022@163.com (P.Z.); yangxiaoqin@swfu.edu.cn (X.Y.)
† These authors contributed equally to this work.

Total 18 pages, 8 Figures and 3 Tables

Conents of the Figures and the Tables

Figure S1. Melanin biosynthesis process

Figure S2. Chemical structures of *beta*-arbutin (1) and 6'-*O*-caffeoylarbutin (2)

Figure S3. ^{1}H NMR (A) and ^{13}C NMR (B) spectra of *beta*-arbutin

Figure S4. ^{1}H NMR (A) and ^{13}C NMR (B) spectra of 6'-*O*-caffeoylarbutin

Figure S5. Fluorescence quenching effect of 6'-*O*-caffeoylarbutin on mTyr

Figure S6. The docking of kojic acid with amino acid residue of pocket of mTyr.

Figure S7. Molecular dynamics (MD) simulation of 6'-*O*-caffeoylarbutin with mTyr

Figure S8. The browning inhibitory effect of 6'-*O*-caffeoylarbutin on apple juice

Table S1. mTyr inhibitory activities of 6'-*O*-caffeoylarbutin

Table S2. Observation of hydrogen bonds in the 6'-*O*-caffeoylarbutin-2Y9X protein complex obtained by molecular docking

Table S3. The results acute test of 6'-*O*-caffeoylarbutin in mice (n=20)

Table S4. Effect of 6'-*O*-caffeoylarbutin on body weight in mice

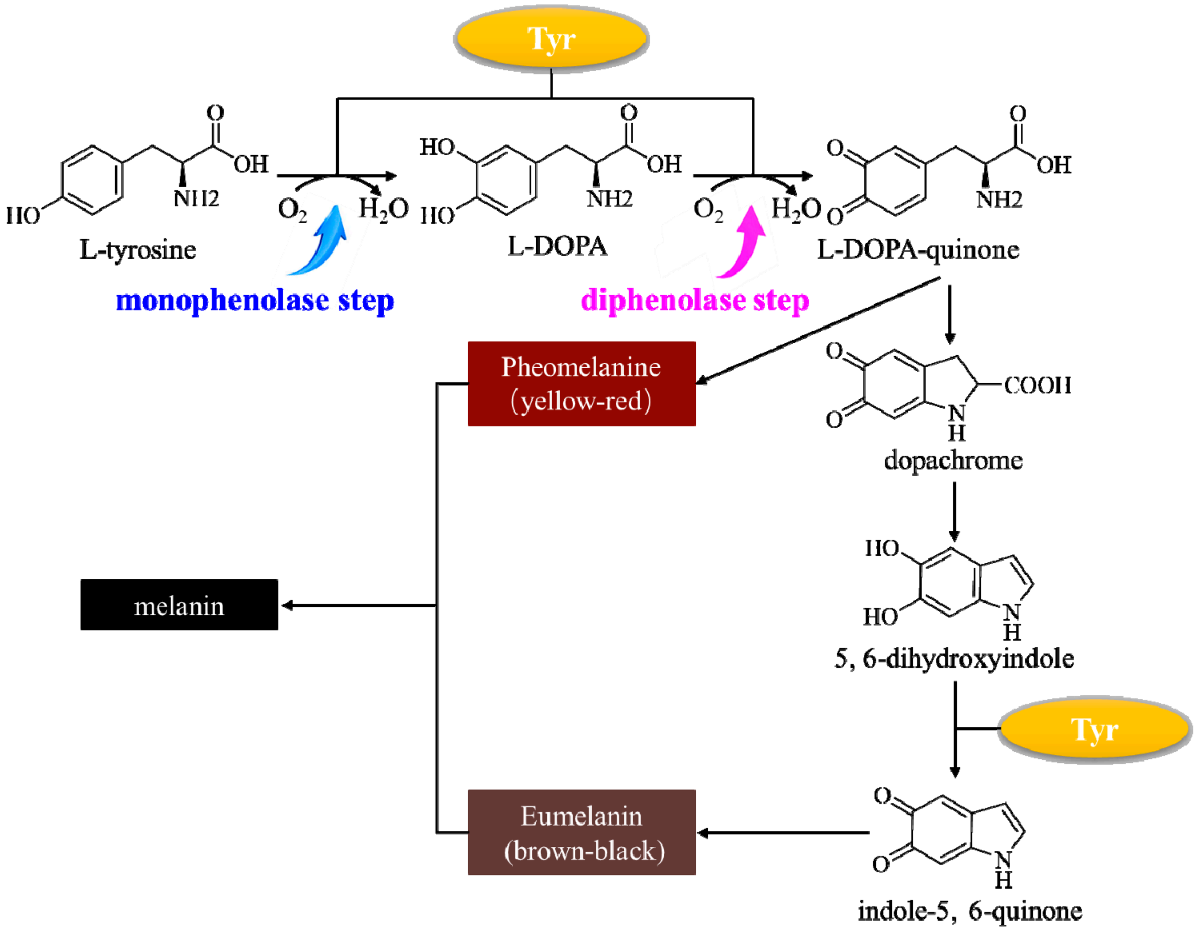

Figure S1. Melanin biosynthesis process

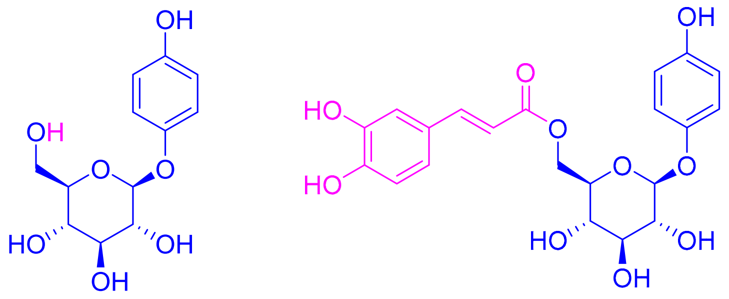

1 **2**

Figure S2. Chemical structures of *beta*-arbutin (1) and 6'-*O*-caffeoylarbutin (2)

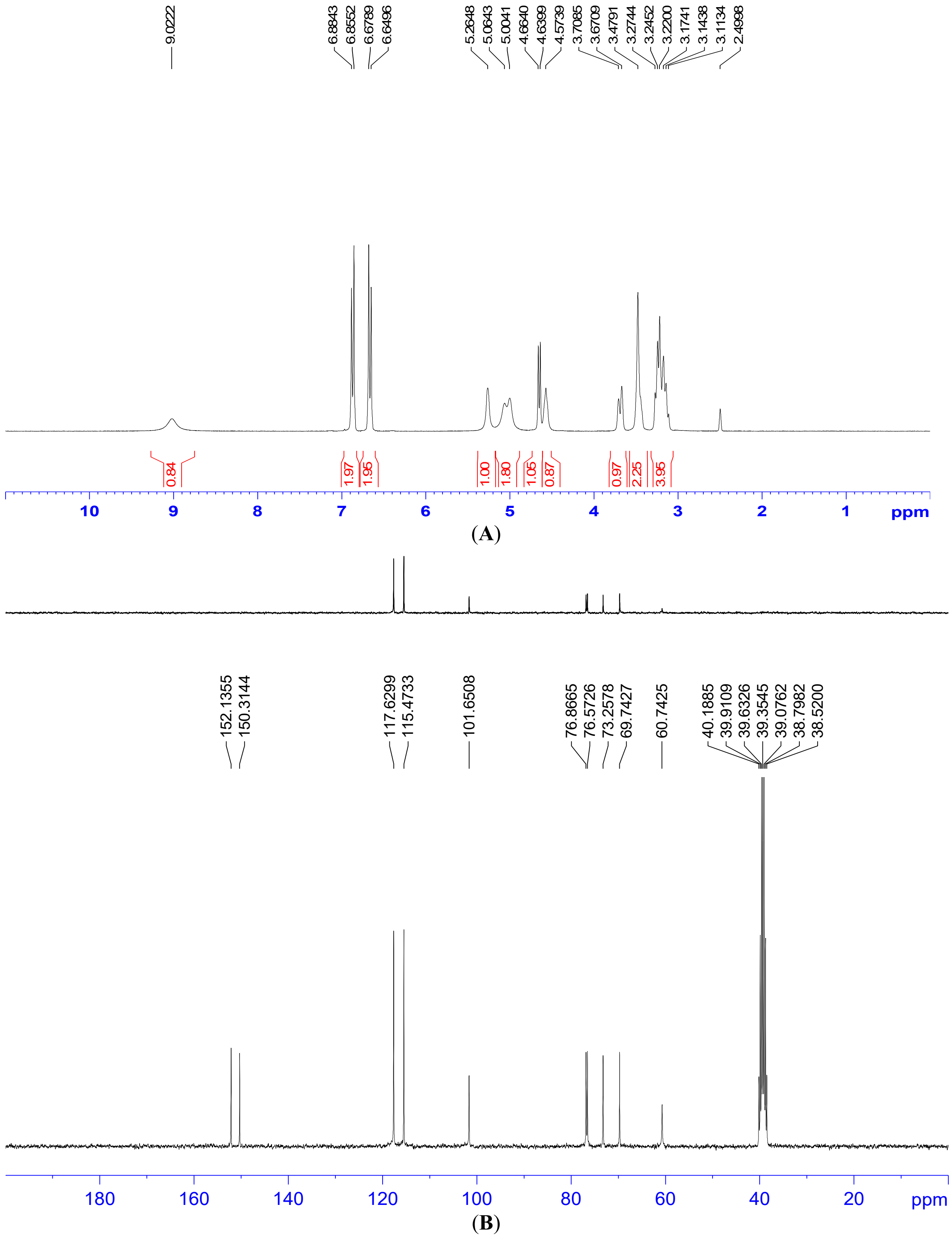

Figure S3. ^{1}H NMR (**A**) and ^{13}C NMR (**B**) spectra of *beta*-arbutin

^{1}H NMR and ^{13}C NMR assignments for *beta*-arbutin as follows:

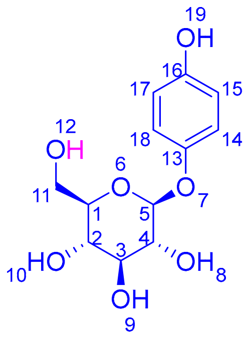

1

beta-arbutin (**1**), white solid, ^{1}H NMR (300 MHz, DMSO) δ_H: 9.02 (H, s, 19-H), 6.86 (2H, d, J = 8.6 Hz, H-15, H-17), 6.66 (2H, d, J = 8.6 Hz, H-14, H-18), 5.26 (1H, s, H-5), 5.03(2H, d, J = 18.0 Hz, H-8, H-10), 4.64 (1H, d, J = 7.2 Hz, H-9), 4.57(1H, s,H-12), 3.69(1H, d, J = 11.7 Hz, H-4), 3.48 (2H, s, H-2, H-3), 3.11-3.27 (3H, m, H-1, H-11); ^{13}C NMR (75 MHz, DMSO), δ_C: 152.1 (C-13), 150.3 (C-16), 117.6 (C-14, C-18), 115.5 (C-15, C-17), 101.7 (C-5), 76.9 (C-1), 76.6 (C-3), 73.3 (C-4), 69.7 (C-2), 60.7 (C-11).

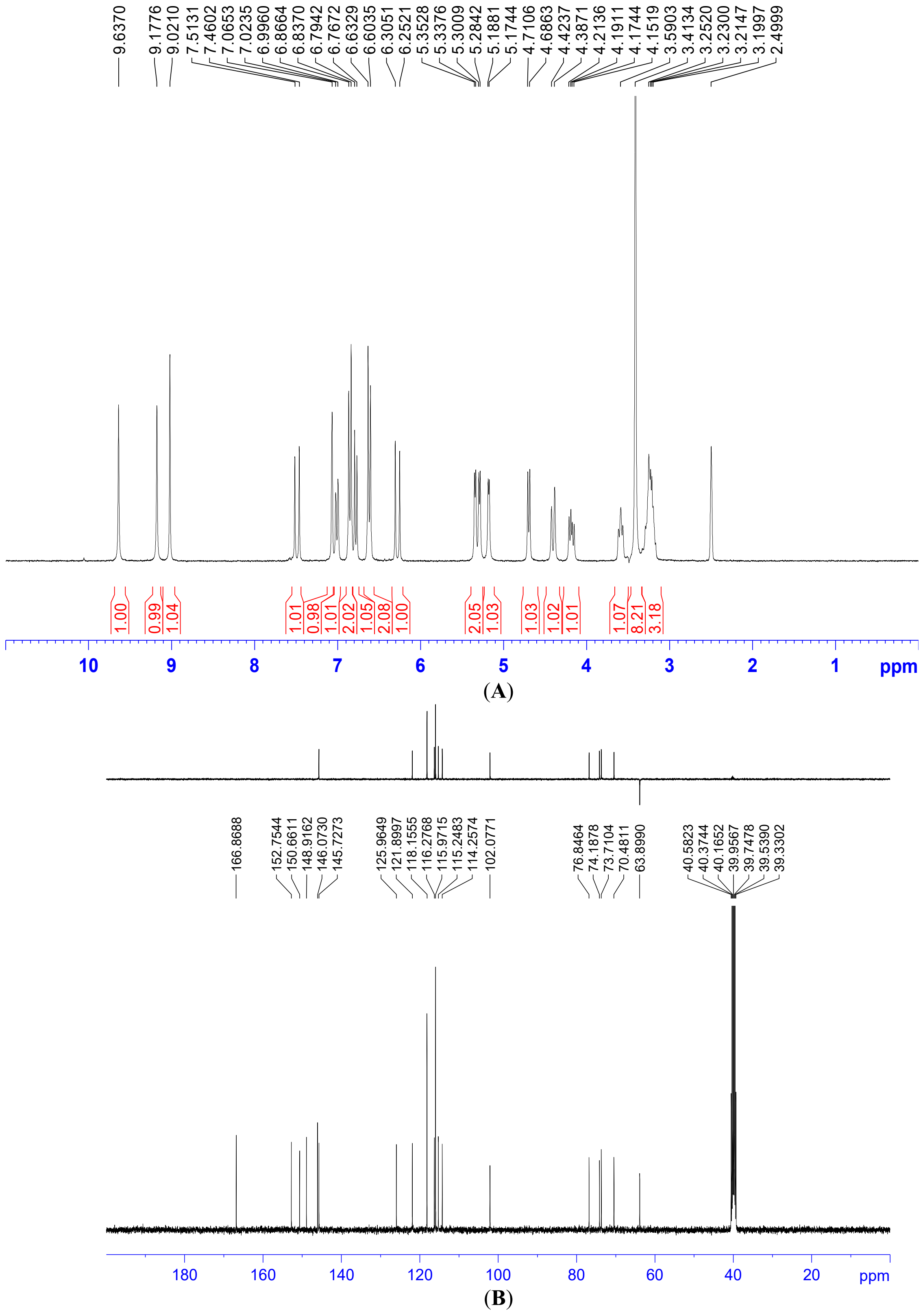

Figure S4. ^{1}H NMR (**A**) and ^{13}C NMR (**B**) for 6'-*O*-caffeoylarbutin

^{1}H NMR and ^{13}C NMR assignments for 6'-*O*-caffeoylarbutin as follows:

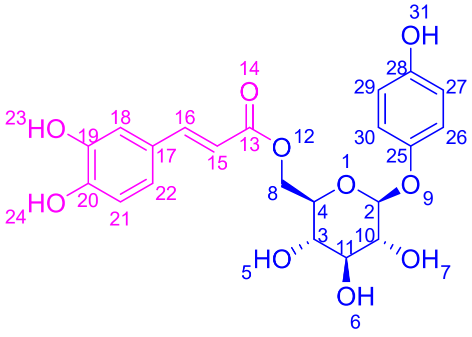

2

6'-*O*-caffeoylarbutin (**2**), colorless needles, ^{1}H NMR (300 MHz, DMSO) δ_{H}: 9.64 (1H, s, H-23), 9.18 (1H, s, H-24), 9.02 (1H, s,H-31), 7.48 (1H, d, *J* = 15.9, H-16), 7.05(1H, s, H-18), 7.01 (1H, d, *J* = 1.8 Hz, H-21), 6.84 (1H, d, *J* = 8.3 Hz, H-22), 6.78 (2H, d, *J* = 8.1 Hz, H-27/29), 6.62 (2H, d, *J* = 8.1 Hz, H-26/30), 6.28 (1H, d, *J* = 15.9 Hz, H-15), 5.34 (2H, m, *J* = 8.1 Hz, H-8), 5.29 (1H, d, *J* = 4.2 Hz, H-2), 4.70 (1H, d, *J* = 7.3 Hz, H-10), 4.39 (1H, d, *J* = 11.1 Hz, H-11), 4.20 (1H, m, H-3), 3.59 (1H, t, H-4); ^{13}C NMR (75 MHz, DMSO), δ_{C}: 166.9 (C-13), 152.8 (C-25), 150.7 (C-28), 148.9 (C-20), 146.1 (C-19), 145.7 (C-16), 126.0 (C-17), 121.9 (C-22), 118.2 (C-26/30), 116.3 (C-15), 116.0 (C-27/29), 115.2 (C-18), 114.3 (C-21), 102.1 (C-2), 76.8 (C-4), 74.2 (C-11), 73.7 (C-10), 70.5 (C-3), 63.9 (C-8).

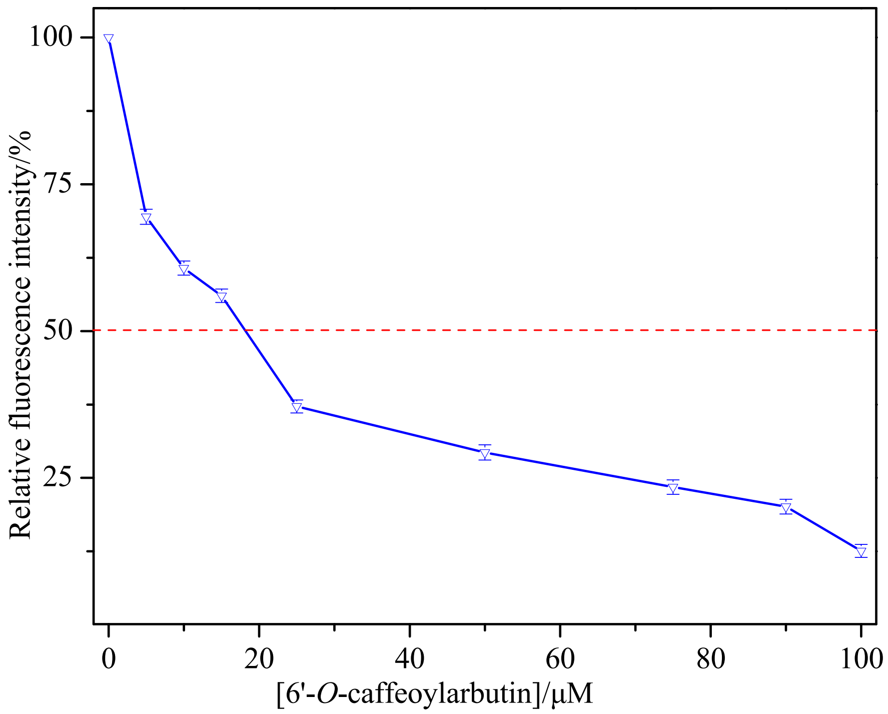

100
75
50
25
Relative fluorescence intensity/%
0
20
40
60
80
100
[6'-O-caffeoylarbutin]/μM

(A)

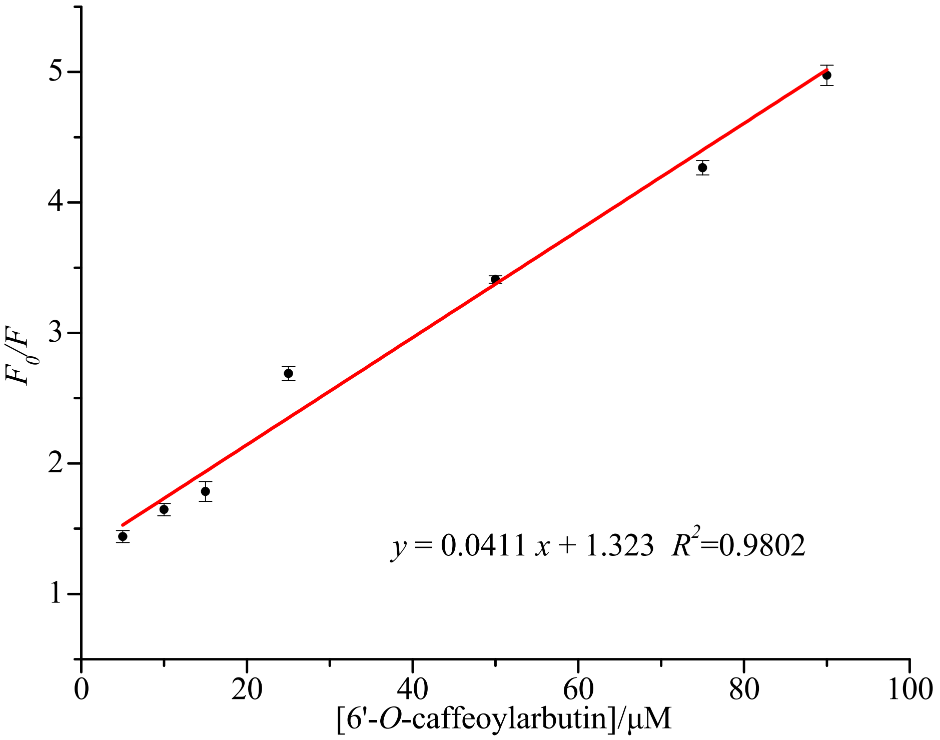

5
4
3
2
1
F_0/F
$y = 0.0411\ x + 1.323\ \ R^2=0.9802$
0
20
40
60
80
100
[6'-O-caffeoylarbutin]/μM

(**B**)

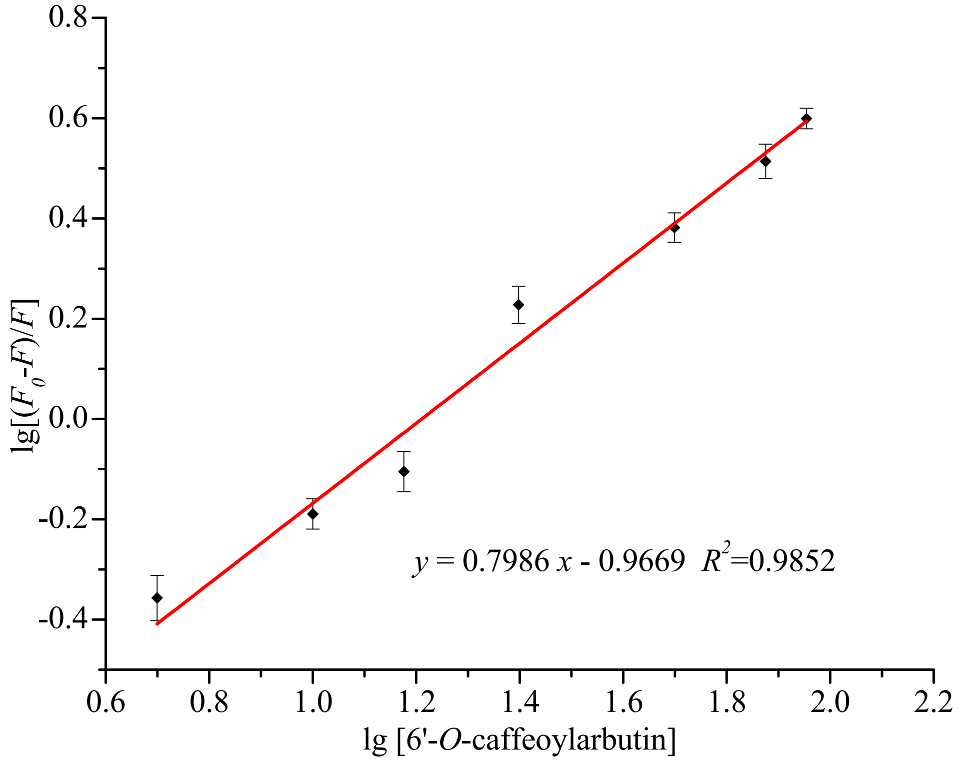

(**C**)

Figure S5. Fluorescence quenching effect of 6'-*O*-caffeoylarbutin on mTyr

(**A**). Relative fluorescence intensity of mTyr at different concentrations of 6'-*O*-caffeoylarbutin. (**B**). Stem-Volmer plot illustrating the fluorescence quenching of mTyr. C. Plot of lg [(F_0–F)/F] against lg [6'-*O*-caffeoylarbutin] for mTyr.

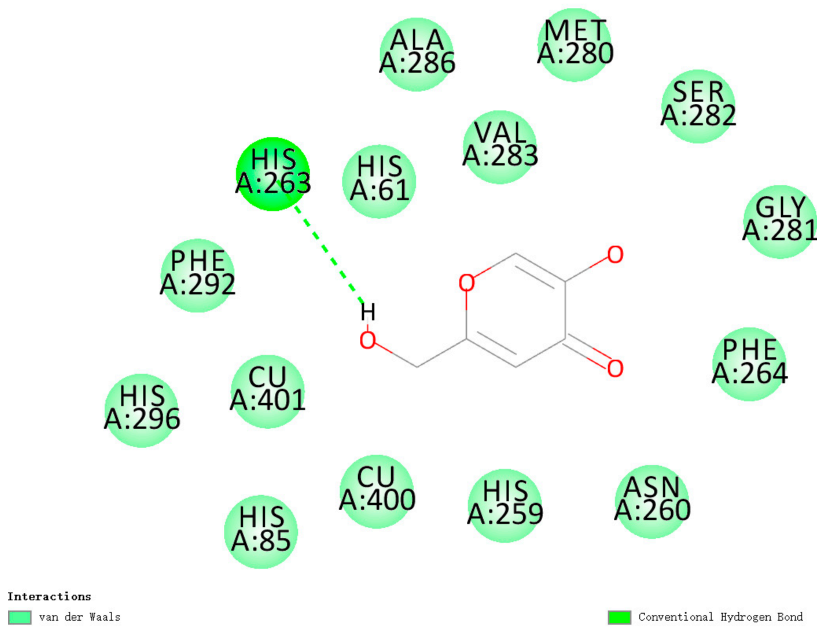

Figure S6. The docking of kojic acid with amino acid residue of pocket of mTyr.

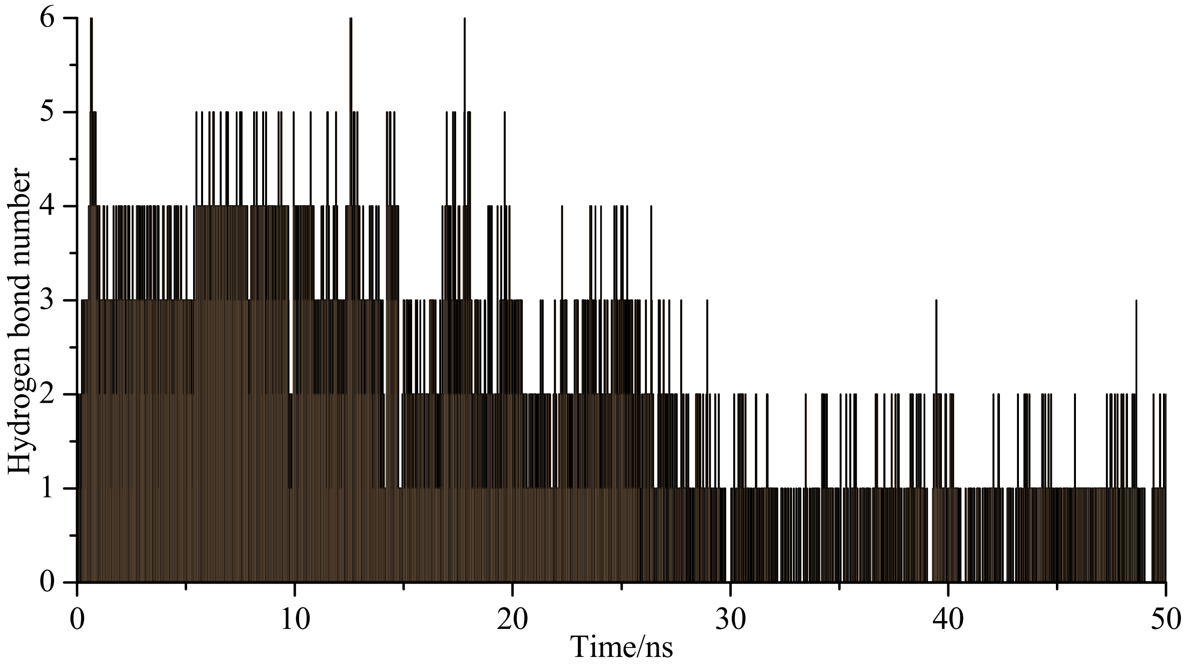

(**A**)

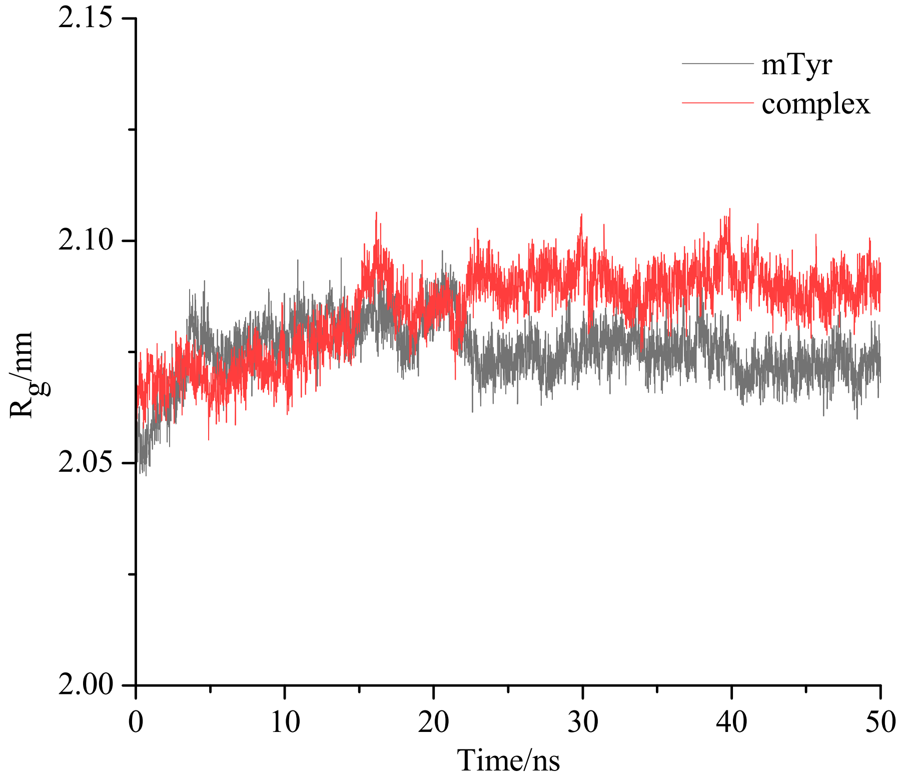

(**B**)

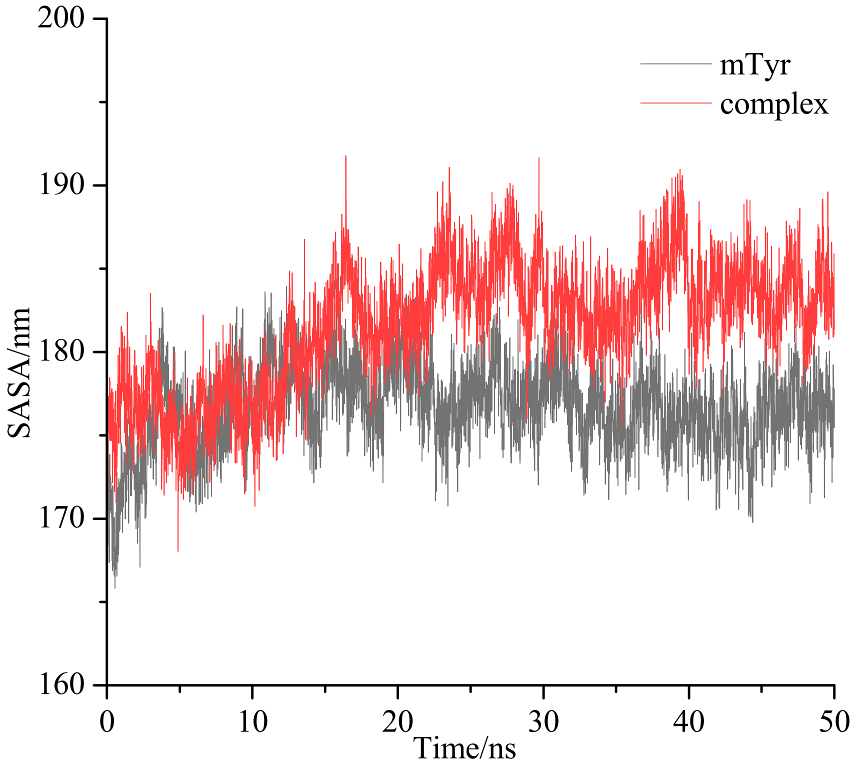

(**C**)

Figure S7. Molecular dynamics (MD) simulation of 6'-*O*-caffeoylarbutin with mTyr

(**A**). The number of hydrogen bonds analysis. (**B**). Radius of gyration (Rg) change curve. (**C**). Solvent accessible surface area (SASA) change curve.

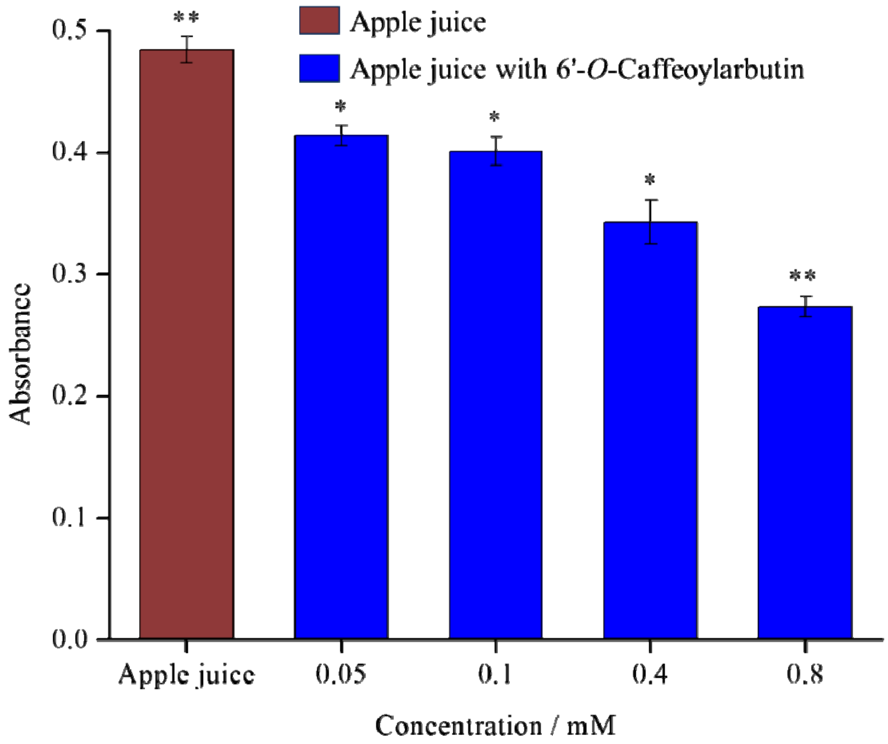

Figure S8. The browning inhibitory effect of 6'-*O*-caffeoylarbutin on apple juice

Table S1. mTyr inhibitory activities of 6'-*O*-caffeoylarbutin

Compound	IC_{50} [a]	
	Substrate [L-Tyrosine]	Substrate [L-DOPA]
6'-*O*-Caffeoylarbutin	1.1±0.1 μM	95.2±1.1μM
beta-Arbutin	8.4 mM	-

[a]IC_{50} values of all compounds are used as reference (Funayama et al., 1995 https://doi.org/10.1271/bbb.59.143; Liu et al., 2023 https://doi.org/10.13386/j.issn1002-0306.2022070024) and represent the concentration that caused 50% enzyme activity loss.

Table S2. Observation of hydrogen bonds in the 6'-*O*-caffeoylarbutin-2y9x protein complex obtained by molecular docking

Amino acid species	6'-*O*-caffeoylarbutin	Atoms forming hydrogen bonds
Glu 322	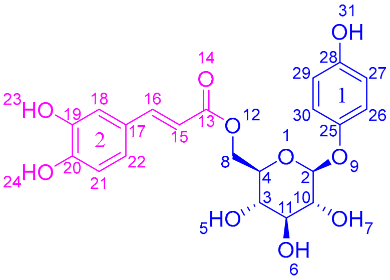	hydrogen atom at position 23
His 85		hydrogen atom at position 7
Val 283		oxygen atom at position 7
Gly 281		oxygen atom at position 12
His 244		benzene ring 1

Table S3. The results acute test of 6'-*O*-caffeoylarbutin in mice (n=20)

Group	Number of mice	Final dose ($mg \cdot kg^{-1}$)	Death toll（♀/♂）	Death rate (%)
Vehicle	20	-	0/0	0
CA-1	20	28056	3/2	25
CA-2	20	23848	0/1	5
CA-3	20	20272	0/0	0

Table S4. Effect of 6'-*O*-caffeoylarbutin on body weight in mice

Group	Gender	Initial body weight	After the delivery				Body weight gain (g)
			1st day	3rd day	7th day	14th day	
Vehicle	Female	21.37±0.79	23.22±1.12	24.30±1.05	25.06±0.61	27.42±1.07	2.36±1.22
	Male	30.89±0.91	33.42±1.36	33.17±1.10	35.63±1.07	38.00±1.88	2.37±1.87
CA-1	Female	21.34±0.66	22.26±0.97	24.28±1.18	24.88±1.09	27.10±1.39	2.22±0.62
	Male	30.86±0.73	30.75±2.11**	33.62±2.35	35.25±2.53	37.90±3.13	2.65±1.01
CA-2	Female	21.20±0.60	21.58±1.21**	23.05±0.45**	23.59±0.50**	25.47±1.36	1.88±1.17
	Male	29.96±0.94	30.91±0.87**	33.07±1.24	33.88±1.71	37.26±2.19	3.38±1.23
CA-3	Female	21.27±0.58	22.21±0.87	23.36±0.66*	24.21±0.91 *	26.62±1.54	2.41±1.51
	Male	30.32±0.88	30.89±1.57**	33.88±1.41	35.33±1.44	36.78±3.53	1.45±3.11

Statistical analysis: */** had a significant difference compared with the Vehicle group (p<0.05/0.01).
